# *Bifidobacterium breve* Sepsis in Child with High-Risk Acute Lymphoblastic Leukemia

**DOI:** 10.3201/eid2109.150097

**Published:** 2015-09

**Authors:** Simona Lucija Avcin, Marko Pokorn, Lidija Kitanovski, Manica Mueller Premru, Janez Jazbec

**Affiliations:** University Medical Centre, Ljubljana, Slovenia (S.L. Avcin, M. Pokorn, L. Kitanovski, J. Jazbec);; Institute for Microbiology and Immunology, Ljubljana (M.M. Premru)

**Keywords:** *Bifidobacterium breve*, sepsis, leukemia, bacteria, probiotics

**To the Editor:** Patients with cancer often consume probiotics as part of their diet, although therapeutic use of probiotics is not recommended because of their potential invasiveness. In a recent review, 5 cases of probiotic treatment–related bacteremia were identified in oncology patients, although no cases of invasive *Bifidobacterium* spp. infection were included (*1*). We describe a case of *B. breve* sepsis in a child with Philadelphia chromosome–positive acute B-cell lymphoblastic leukemia.

The patient was a previously healthy 2-year-old boy who had no history of immunodeficiency and whose leukocyte counts had been within reference ranges during check-up visits before his diagnosis. After leukemia was diagnosed, chemotherapy was started (prednisone, vincristine, doxorubicin, and L-asparaginase). During the second week of treatment, the boy experienced abdominal discomfort and constipation. Two weeks later, his condition worsened; he refused food, his abdomen was distended, and he had colicky pain. Thickened intestinal wall and fecal masses were seen ultrasonographically. Twelve hours later he became hypotensive. Laboratory test results showed severe neutropenia and increased inflammatory markers (Figure). Two aerobic and anaerobic blood culture samples were collected from a central venous line (implantable venous access system) in a 30-minute span, and treatment with piperacillin/tazobactam, vancomycin, and gentamicin was empirically initiated according to local recommendations for pediatric febrile neutropenia with shock. Both anaerobic blood cultures were positive. Gram-positive, irregular rods with bifid and branching forms without spores grew anaerobically on blood agar with hemin and vitamin K after 48 hours of incubation and were identified as *B. breve* by matrix-assisted laser desorption/ionization time-of-flight mass spectrometry (Bruker Daltonics, Billerica, MA, USA). The bacteria were susceptible to penicillin (MIC 0.250 μg/mL), ampicillin, amoxicillin/clavulanic acid, piperacillin/tazobactam (MIC 0.125 μg/mL), imipenem, and clindamycin but not metronidazole. Gentamicin and vancomycin were discontinued, and piperacillin/tazobactam was replaced by penicillin (Figure). The patient stayed afebrile, and his neutropenia resolved. A blood culture taken on the eighth day of antimicrobial drug treatment was negative, and the central venous line was not replaced at that time. Bowel movement normalized and was maintained. We reviewed the ingredients of the food that the child received and documented the presence of *Lactobacillus* spp. and *B. longum* but not *B. breve*.

**Figure:**
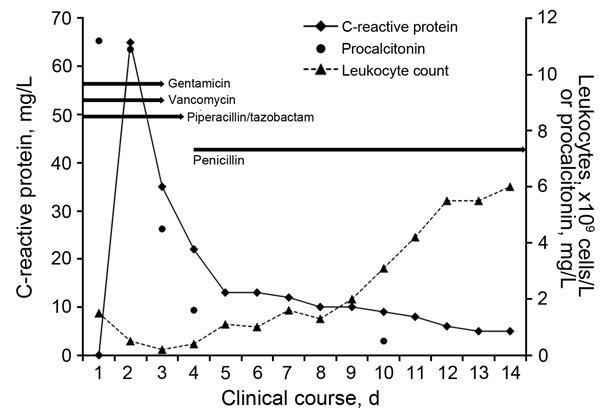
Schematic presentation of leukocyte count, C-reactive protein, and procalcitonin serum levels in clinical course of *Bifidobacterium breve* sepsis. Arrows indicate the name and duration of each antimicrobial drug treatment.

Some probiotics are part of the normal intestinal microbiota and rarely cause invasive infections (*2*). Although *Bifidobacterium* spp. is infrequently associated with infections (<5% of anaerobic isolates), it occasionally causes serious illness. On the rare occasions when it is isolated from patients with bloodstream infections, it is usually isolated along with other causative agents. The number of reported deaths associated with anaerobic nonsporulating gram-positive rods is low (*3*). In this patient, abdominal symptoms coincided with 2 blood cultures that yielded *B. breve*. We assume bacteria translocated through the distended colonic wall during chemotherapy-induced neutropenia and believe that the blood culture isolate was not a contaminant because it was isolated from 2 samples taken in a 30-min span. It is the practice at our institution not to take peripheral blood cultures simultaneously because doing so does not increase diagnostic accuracy.

We found 1 description in the literature of *B. breve* septicemia in a neonate with omphalocele who had received probiotic therapy (*4*). In a review of *Bifidobacterium* spp. isolates during 2000–2007 in 2 US hospitals, *B. breve* was isolated from blood culture from 3 adult patients (*5*). Two of these infections were associated with ileal resection or peritonitis and 1 with decubitus ulcers. No data on antimicrobial drug treatment were available. *Bifidobacterium* spp. sepsis was reported in an infant of extremely low birthweight 10 days after probiotic supplementation who recovered after antimicrobial drug therapy, although stenosis of the colon developed 6 weeks later (*6*). Blood culture grew *B. longum* and *B. infantis*, which were probiotic strains. Apart from 1 case of sepsis caused by *B. longum* associated with acupuncture in a 19-year-old healthy patient (*7*), we did not find other reports of invasive *Bifidobacterium* spp. infections. 

Because neutropenic episodes, even with bowel involvement, are common during treatment for cancer (*8*), no reason to promote therapeutic use of probiotics has been proven. Probiotics can cause substantial bacterial overgrowth when stimulating factors are present. In our opinion, avoiding fecal impaction is crucial for preventing colonic bacterial overgrowth and minimizes the chance that bacteria will translocate and cause invasive infection. Nutritional recommendations for a neutropenic diet for children are still debated. The problem is not probiotic therapy but rather fermented food products to which small amounts of probiotics are added. After we reviewed the literature, we did not find enough data to safely recommend the use of these products in children receiving chemotherapy (*9*). Nevertheless, probiotic therapy is recommended for many immunocompromised patients, such as preterm infants and persons with chronic inflammatory bowel disease (*10*). We believe that this case of *B. breve* sepsis in an oncology patient underscores the invasive potential of probiotics.
